# Supplementary material for: A UMLS-based spell checker for natural language processing in vaccine safety
Source: BMC Med Inform Decis Mak. 2007 Feb 12;7:3. doi: 10.1186/1472-6947-7-3 (PMC1805499; doi:10.1186/1472-6947-7-3)
Supplement: Additional file 13 — RAP application directory. Sets up directory for RAP (RDF application for PHP) source code [file 1472-6947-7-3-S13.gz › rap/doc/phpdoc/__filesource/fsource_rdql__rdqlRdqlDbEngine.php.html]

xml version="1.0" encoding="iso-8859-1"?


File Source for RdqlDbEngine.php


# Source for file RdqlDbEngine.php

Documentation is available at RdqlDbEngine.php

```
1. <?php

3. // ----------------------------------------------------------------------------------
4. // Class: RdqlDbEngine
5. // ----------------------------------------------------------------------------------


9. /**
10. * This class performs as RDQL query on a DbModel.
11. *
12. * Provided an rdql query parsed into an array of php variables and constraints
13. * at first the engine generates an sql statement and queries the database for
14. * tuples matching all patterns from the WHERE clause of the given RDQL query.
15. * Subsequently the result set is is filtered with evaluated boolean expressions
16. * from the AND clause of the given RDQL query.
17. *
18. * <BR><BR>History:<UL>
19. * <LI>09-27-2004                                 : Multiple queries bug fixed</LI>
20. * <LI>05-12-2004                 : Bug in the handling of empty Literals fixed.</LI>
21. * <LI>08-29-2003                : Function filterTuples(): some bugs fixed:
22. *                                 - strEqExpr with NE operator
23. *                                 - regExExpr combined with other expr. and negation (!)
24. *                                   e.g. !(?x ~~ "/sth/" && ?x > 5)
25. * <LI>07-27-2003                : First version of this class</LI>
26. *
27. * @version  V0.9.1
28. * @author   Radoslaw Oldakowski <radol@gmx.de>
29. *
30. * @package rdql
31. * @access public
32. */

34. Class RdqlDbEngine extends RdqlEngine {


37. /**
38. * Parsed query variables and constraints.
39. *
40. * @var     array   ['selectVars'][] = ?VARNAME
41. *                   ['sources'][] = URI
42. *                   ['patterns'][]['subject']['value'] = VARorURI
43. *                                 ['predicate']['value'] = VARorURI
44. *                                 ['object']['value'] = VARorURIorLiterl
45. *                                           ['is_literal'] = boolean
46. *                                           ['l_lang'] = string
47. *                                           ['l_dtype'] = string
48. *                   ['filters'][]['string'] = string
49. *                                ['evalFilterStr'] = string
50. *                                ['reqexEqExprs'][]['var'] = ?VARNAME
51. *                                                  ['operator'] = (eq | ne)
52. *                                                  ['regex'] = string
53. *                                ['strEqExprs'][]['var'] = ?VARNAME
54. *                                                ['operator'] = (eq | ne)
55. *                                                ['value'] = string
56. *                                                ['value_type'] = ('variable' | 'URI' | 'Literal')
57. *                                                ['value_lang'] = string
58. *                                                ['value_dtype'] = string
59. *                                ['numExpr']['vars'][] = ?VARNAME
60. *                          ( [] stands for an integer index - 0..N )
61. * @access    private
62. */
63. var $parsedQuery;


66. /**
67. * When an RDQL query is performed on a DbModel, in first step the engine searches
68. * in database for triples matching the Rdql-WHERE clause. A recordSet is returned.
69. * $rsIndexes maps select and filter variables to their corresponding indexes
70. * in the returned recordSet.
71. *
72. * @var     array [?VARNAME]['value'] = integer
73. *                           ['nType'] = integer
74. *                           ['l_lang'] = integer
75. *                           ['l_dtype'] = integer
76. * @access    private
77. */
78. var $rsIndexes;


81. /**
82. * Perform an RDQL Query on the given DbModel.
83. *
84. * @param   object  DbModel $dbModel
85. * @param   array   &$parsedQuery  (the same format as $this->parsedQuery)
86. * @param   boolean $returnNodes
87. * @return  array   [][?VARNAME] = object Node  (if $returnNodes = TRUE)
88. *       OR  array   [][?VARNAME] = string
89. * @access  public
90. */
91. function & queryModel(&$dbModel, &$parsedQuery, $returnNodes = TRUE) {

93. $this->parsedQuery = &$parsedQuery;

95. $sql = $this->generateSql($dbModel->modelID);
96. $recordSet =& $dbModel->dbConn->execute($sql);
97. $queryResult = $this->filterQueryResult($recordSet);

99. if ($returnNodes)
100. return $this->toNodes($queryResult);
101. else
102. return $this->toString($queryResult);
103. }


106. /**
107. * Generate an SQL string to query the database for tuples matching all patterns
108. * of $parsedQuery.
109. *
110. * @param   integer $modelID
111. * @return  string
112. * @access    private
113. */
114. function generateSql($modelID) {

116. $sql  = $this->generateSql_SelectClause();
117. $sql .= $this->generateSql_FromClause();
118. $sql .= $this->generateSql_WhereClause($modelID);
119. return $sql;
120. }


123. /**
124. * Generate SQL SELECT clause.
125. *
126. * @return  string
127. * @throws  PHPError
128. * @access    private
129. */
130. function generateSql_SelectClause() {

132. $sql_select = 'SELECT';
133. $index = 0;
134. $this->rsIndexes = array();

136. foreach ($this->parsedQuery['selectVars'] as $var)
137. $sql_select .= $this->_generateSql_SelectVar($var, $index);

139. if (isset($this->parsedQuery['filters'])) {
140. foreach ($this->parsedQuery['filters'] as $n => $filter) {

142. // variables from numeric expressions
143. foreach ($filter['numExprVars'] as $numVar)
144. $sql_select .= $this->_generateSql_SelectVar($numVar, $index);

146. // variables from regex equality expressions
147. foreach ($filter['regexEqExprs'] as $regexEqExpr)
148. $sql_select .= $this->_generateSql_SelectVar($regexEqExpr['var'], $index);

150. // variables from string equality expressions
151. foreach ($filter['strEqExprs'] as $strEqVar)
152. $sql_select .= $this->_generateSql_SelectVar($strEqVar['var'], $index);
153. }
154. }

156. return rtrim($sql_select, " , ");
157. }


160. /**
161. * Generate SQL FROM clause
162. *
163. * @return  string
164. * @access    private
165. */
166. function generateSql_FromClause() {

168. $sql_from = ' FROM';
169. foreach ($this->parsedQuery['patterns'] as $n => $v)
170. $sql_from .= ' statements s' .($n+1) .' , ';

172. return rtrim($sql_from, ' , ');
173. }


176. /**
177. * Generate an SQL WHERE clause
178. *
179. * @param   integer $modelID
180. * @return  string
181. * @access    private
182. */
183. function generateSql_WhereClause($modelID) {

185. $sql_where = ' WHERE';
186. $count_patterns = count($this->parsedQuery['patterns']);
187. foreach ($this->parsedQuery['patterns'] as $n => $pattern) {
188. $sql_where .= ' s' .($n+1) .'.modelID=' .$modelID .' AND';
189. foreach ($pattern as $key => $val_1)
190. if ($val_1['value'] && $val_1['value']{0}=='?') {
191. $sql_tmp = ' s' .($n+1) .'.' .$key .'=';
192. // find internal bindings
193. switch ($key) {
194. case 'subject':
195. if ($pattern['subject']['value'] == $pattern['predicate']['value'])
196. $sql_where .= $sql_tmp .'s' .($n+1) .'.predicate AND';
197. elseif ($pattern['subject']['value'] == $pattern['object']['value'])
198. $sql_where .= $sql_tmp .'s' .($n+1) .'.object AND';
199. break;
200. case 'predicate':
201. if ($pattern['predicate']['value'] == $pattern['object']['value'])
202. $sql_where .= $sql_tmp .'s' .($n+1) .'.object AND';
203. }
204. // find external bindings
205. for ($i=$n+1; $i<$count_patterns; $i++)
206. foreach ($this->parsedQuery['patterns'][$i] as $key2 => $val_2)
207. if ($val_1['value']==$val_2['value']) {
208. $sql_where .= $sql_tmp .'s' .($i+1) .'.' .$key2 .' AND';
209. break 2;
210. }
211. }else {
212. $sql_where .= ' s' .($n+1) .'.' .$key ."='" .$val_1['value'] ."' AND";
213. if ($key == 'object' && isset($val_1['is_literal'])) {
214. $sql_where .= ' s' .($n+1) .".object_is='l' AND";
215. $sql_where .= ' s' .($n+1) .".l_datatype='" .$val_1['l_dtype'] ."' AND";
216. $sql_where .= ' s' .($n+1) .".l_language='" .$val_1['l_lang'] ."' AND";
217. }
218. }
219. }
220. return rtrim($sql_where, ' AND');
221. }


224. /**
225. * Filter tuples containing variables matching all patterns from the WHERE clause
226. * of an RDQL query. As a result of a database query using ADOdb these tuples
227. * are returned as an ADORecordSet object, which is then passed to this function.
228. *
229. * @param   object ADORecordSet &$recordSet
230. * @return  array  [][?VARNAME]['value']   = string
231. *                              ['nType']   = string
232. *                              ['l_lang']  = string
233. *                              ['l_dtype'] = string
234. * @access    private
235. */
236. function filterQueryResult(&$recordSet) {

238. $queryResult=array();

240. if (isset($this->parsedQuery['filters'])) {

242. while (!$recordSet->EOF) {

244. foreach ($this->parsedQuery['filters'] as $filter) {

246. $evalFilterStr = $filter['evalFilterStr'];

248. // evaluate regex equality expressions of each filter
249. foreach ($filter['regexEqExprs'] as $i => $expr) {
250. preg_match($expr['regex'], $recordSet->fields[$this->rsIndexes[$expr['var']]['value']], $match);
251. $op = substr($expr['operator'], 0,1);
252. if (($op != '!' && !isset($match[0])) || ($op == '!' && isset($match[0])))
253. $evalFilterStr = str_replace("##RegEx_$i##", 'FALSE', $evalFilterStr);
254. else
255. $evalFilterStr = str_replace("##RegEx_$i##", 'TRUE', $evalFilterStr);
256. }

258. // evaluate string equality expressions
259. foreach ($filter['strEqExprs'] as $i => $expr) {

261. $exprBoolVal = 'FALSE';

263. switch ($expr['value_type']) {

265. case 'variable':
266. if (($recordSet->fields[$this->rsIndexes[$expr['var']]['value']] ==
267. $recordSet->fields[$this->rsIndexes[$expr['value']]['value']] &&
268. $expr['operator'] == 'eq') ||
269. ($recordSet->fields[$this->rsIndexes[$expr['var']]['value']] !=
270. $recordSet->fields[$this->rsIndexes[$expr['value']]['value']] &&
271. $expr['operator'] == 'ne'))

273. $exprBoolVal = 'TRUE';
274. break;

276. case 'URI':

278. if (isset($this->rsIndexes[$expr['var']]['nType']) &&
279. $recordSet->fields[$this->rsIndexes[$expr['var']]['nType']] == 'l') {

281. if ($expr['operator'] == 'ne')
282. $exprBoolVal = 'TRUE';
283. break;
284. }

286. if (($recordSet->fields[$this->rsIndexes[$expr['var']]['value']] ==
287. $expr['value'] && $expr['operator'] == 'eq') ||
288. ($recordSet->fields[$this->rsIndexes[$expr['var']]['value']] !=
289. $expr['value'] && $expr['operator'] == 'ne'))
290. $exprBoolVal = 'TRUE';
291. break;

293. case 'Literal':

295. if (!isset($this->rsIndexes[$expr['var']]['nType']) ||
296. $recordSet->fields[$this->rsIndexes[$expr['var']]['nType']] != 'l') {

298. if ($expr['operator'] == 'ne')
299. $exprBoolVal = 'TRUE';
300. break;
301. }

303. $filterLiteral= new Literal($expr['value'],$expr['value_lang']);
304. $filterLiteral->setDatatype($expr['value_dtype']);

306. $resultLiteral=new Literal($recordSet->fields[$this->rsIndexes[$expr['var']]['value']]);
307. $resultLiteral->setDatatype($recordSet->fields[$this->rsIndexes[$expr['var']]['l_dtype']]);
308. $resultLiteral->setLanguage($recordSet->fields[$this->rsIndexes[$expr['var']]['l_lang']]);

310. $equal=$resultLiteral->equals($filterLiteral);

312. if (($equal && $expr['operator'] == 'eq') ||
313. (!$equal && $expr['operator'] == 'ne'))
314. $exprBoolVal = 'TRUE';
315. else
316. $exprBoolVal = 'FALSE';

318. }

320. $evalFilterStr = str_replace("##strEqExpr_$i##", $exprBoolVal, $evalFilterStr);
321. }

323. // evaluate numerical expressions
324. foreach ($filter['numExprVars'] as $varName) {
325. $varValue = "'" .$recordSet->fields[$this->rsIndexes[$varName]['value']] ."'";
326. $evalFilterStr = str_replace($varName, $varValue, $evalFilterStr);
327. }

329. eval("\$filterBoolVal = $evalFilterStr; \$eval_filter_ok = TRUE;");
330. if (!isset($eval_filter_ok))
331. trigger_error(RDQL_AND_ERR ."'" .htmlspecialchars($filter['string']) ."'", E_USER_ERROR);

333. if (!$filterBoolVal) {
334. $recordSet->MoveNext();
335. continue 2;
336. }

338. }
339. $queryResult[] = $this->_convertRsRowToQueryResultRow($recordSet->fields);
340. $recordSet->MoveNext();
341. }

343. }else
344. while (!$recordSet->EOF) {
345. $queryResult[] = $this->_convertRsRowToQueryResultRow($recordSet->fields);
346. $recordSet->MoveNext();
347. }
348. return $queryResult;
349. }


352. /**
353. * Serialize variable values of $queryResult to string.
354. *
355. * @param   array  &$queryResult [][?VARNAME]['value']   = string
356. *                                            ['nType']   = string
357. *                                            ['l_lang']  = string
358. *                                            ['l_dtype'] = string
359. * @return  array  [][?VARNAME] = string
360. * @access    private
361. */
362. function toString(&$queryResult) {

364. // if a result set is empty return only variable sames
365. if (count($queryResult) == 0) {
366. foreach ($this->parsedQuery['selectVars'] as $selectVar)
367. $res[0][$selectVar] = NULL;
368. return $res;
369. }

371. $res = array();
372. foreach ($queryResult as $n => $var)
373. foreach ($var as $varname => $varProperties)
374. if ($varProperties['nType'] == 'r' || $varProperties['nType'] == 'b')
375. $res[$n][$varname] = '<' .$varProperties['value'] .'>';
376. else {
377. $res[$n][$varname] = '"' .$varProperties['value'] .'"';
378. if ($varProperties['l_lang'] != NULL)
379. $res[$n][$varname] .= ' (xml:lang="' .$varProperties['l_lang'] .'")';
380. if ($varProperties['l_dtype'] != NULL)
381. $res[$n][$varname] .= ' (rdf:datatype="' .$varProperties['l_dtype'] .'")';
382. }
383. return $res;
384. }


387. /**
388. * Convert variable values of $queryResult to objects (Node).
389. *
390. * @param   array  &$queryResult [][?VARNAME]['value']   = string
391. *                                            ['nType']   = string
392. *                                            ['l_lang']  = string
393. *                                            ['l_dtype'] = string
394. * @return  array  [][?VARNAME] = object Node
395. * @access    private
396. */
397. function toNodes(&$queryResult) {

399. // if a result set is empty return only variable sames
400. if (count($queryResult) == 0) {
401. foreach ($this->parsedQuery['selectVars'] as $selectVar)
402. $res[0][$selectVar] = NULL;
403. return $res;
404. }

406. $res = array();
407. foreach ($queryResult as $n => $var)
408. foreach ($var as $varname => $varProperties)
409. if ($varProperties['nType'] == 'r')
410. $res[$n][$varname] = new Resource($varProperties['value']);
411. elseif ($varProperties['nType'] == 'b')
412. $res[$n][$varname] = new BlankNode($varProperties['value']);
413. else {
414. $res[$n][$varname] = new Literal($varProperties['value'], $varProperties['l_lang']);
415. if ($varProperties['l_dtype'] != NULL)
416. $res[$n][$varname]->setDataType($varProperties['l_dtype']);
417. }
418. return $res;
419. }


422. /**
423. * Generate a piece of an sql select statement for a variable.
424. * Look first if the given variable is defined as a pattern object.
425. * (So you can select the node type, literal lang and dtype)
426. * If not found - look for subjects and select node label and type.
427. * If there is no result either go to predicates.
428. * Predicates are always resources therefore select only the node label.
429. *
430. * @param   string $varName
431. * @return  string
432. * @access    private
433. */
434. function _generateSql_SelectVar ($varName, &$index) {

436. $sql_select = '';

438. if (array_key_exists($varName, $this->rsIndexes))
439. return NULL;

441. foreach ($this->parsedQuery['patterns'] as $n => $pattern)
442. if ($varName == $pattern['object']['value']) {

444. // select the object label
445. $sql_select .= " s" .++$n .".object as _" .ltrim($varName, "?") ." , ";
446. $this->rsIndexes[$varName]['value'] = $index++;
447. // select the node type
448. $sql_select .= " s" .$n .".object_is , ";
449. $this->rsIndexes[$varName]['nType'] = $index++;
450. // select the object language
451. $sql_select .= " s" .$n .".l_language , ";
452. $this->rsIndexes[$varName]['l_lang'] = $index++;
453. // select the object dtype
454. $sql_select .= " s" .$n .".l_datatype , ";
455. $this->rsIndexes[$varName]['l_dtype'] = $index++;

457. return $sql_select;
458. }

460. foreach ($this->parsedQuery['patterns'] as $n => $pattern)
461. if ($varName == $pattern['subject']['value']) {

463. // select the object label
464. $sql_select .= " s" .++$n .".subject as _" .ltrim($varName, "?") ." , ";
465. $this->rsIndexes[$varName]['value'] = $index++;
466. // select the node type
467. $sql_select .= " s" .$n .".subject_is , ";
468. $this->rsIndexes[$varName]['nType'] = $index++;

470. return $sql_select;
471. }

473. foreach ($this->parsedQuery['patterns'] as $n => $pattern)
474. if ($varName == $pattern['predicate']['value']) {

476. // select the object label
477. $sql_select .= " s" .++$n .".predicate as _" .ltrim($varName, "?") ." , ";
478. $this->rsIndexes[$varName]['value'] = $index++;

480. return $sql_select;
481. }
482. }


485. /**
486. * Converts a single row of ADORecordSet->fields array to the format of
487. * $queryResult array using pointers to indexes ($this->rsIndexes) in RecordSet->fields.
488. *
489. * @param   array  &$record [] = string
490. * @return  array  [?VARNAME]['value']   = string
491. *                            ['nType']   = string
492. *                            ['l_lang']  = string
493. *                            ['l_dtype'] = string
494. * @access    private
495. */
496. function _convertRsRowToQueryResultRow(&$record) {

498. // return only select variables (without conditional variables from the AND clause)
499. foreach ($this->parsedQuery['selectVars'] as $selectVar) {
500. $resultRow[$selectVar]['value'] = $record[$this->rsIndexes[$selectVar]['value']];
501. if (isset($this->rsIndexes[$selectVar]['nType']))
502. $resultRow[$selectVar]['nType'] = $record[$this->rsIndexes[$selectVar]['nType']];
503. // is a predicate then
504. else
505. $resultRow[$selectVar]['nType'] = 'r';

507. if ($resultRow[$selectVar]['nType'] == 'l') {
508. $resultRow[$selectVar]['l_lang'] = $record[$this->rsIndexes[$selectVar]['l_lang']];
509. $resultRow[$selectVar]['l_dtype'] = $record[$this->rsIndexes[$selectVar]['l_dtype']];
510. }
511. }
512. return $resultRow;
513. }

515. } // end: Class RdqlDbEngine

517. ?>
```

Documentation generated on Thu, 7 Jul 2005 13:43:12 +0200 by phpDocumentor 1.3.0RC3
